# Induced Tomato Plant Resistance Against *Tetranychus urticae* Triggered by the Phytophagy of *Nesidiocoris tenuis*

**DOI:** 10.3389/fpls.2018.01419

**Published:** 2018-10-02

**Authors:** Meritxell Pérez-Hedo, Ángela M. Arias-Sanguino, Alberto Urbaneja

**Affiliations:** Instituto Valenciano de Investigaciones Agrarias, Centro de Protección Vegetal y Biotecnología, Valencia, Spain

**Keywords:** two-spotted spider mite, oviposition, jasmonic acid, protein inhibitors, biological control

## Abstract

The zoophytophagous predator *Nesidiocoris tenuis* (Reuter) (Hemiptera: Miridae) is capable of inducing plant defenses in tomato due to its phytophagous behavior. These induced defenses, which include the release of herbivore-induced plant volatiles (HIPVs), have been proven to affect the oviposition behavior and reduce the subsequent performance of some tomato pests. However, the effect of induction of plant defenses by *N. tenuis* on the preference, development, and reproduction of the two-spotted spider mite *Tetranychus urticae* Koch (Acari: Tetranychidae) remains unknown. In this research, *T. urticae* did not show preference for the odor source emitted by intact tomato plants when compared with *N. tenuis*-punctured plants and jasmonic acid (JA) deficient mutant tomato plants. Furthermore, the number of eggs laid by *T. urticae* on intact tomato plants or on *N. tenuis*-punctured plants was similar. However, in a greenhouse experiment conducted to evaluate whether the defense induction mediated by *N. tenuis* had an effect on *T. urticae* the infestation of *T. urticae* was significantly reduced by 35% on those plants previously activated by *N. tenuis* when compared to the control. The expression of a JA-responsive gene that was upregulated and the transcription of the plant protein inhibitor II was higher on activated plants relative to the control. These results can serve as a basis for the development of new management strategies for *T. urticae* based on plant defense mechanisms induced from the phytophagous behavior of *N. tenuis*.

## Introduction

In recent years the use of omnivorous natural enemies in horticultural crops, and in particular, the zoophytophagous predators that can feed on both plant and prey, has given rise to some of the most resounding successes in biological control in Southern Europe (Jacas and Urbaneja, 2009). In sweet pepper, for example, the release and conservation of the predatory mite *Amblyseius swirskii* (Athias-Henriot) (Acari: Phytoseiidae) together with the anthocorid *Orius laevigatus* (Fieber) (Hemiptera: Anthocoridae) allows successful management of populations of the key pests of this crop: the whitefly *Bemisia tabaci* Gennadius and the thrips *Frankliniella occidentalis* (Pergande) (Thysanoptera: Thripidae) (van der Blom et al., 1997, 2009; Sanchez et al., 2000; Calvo et al., 2009, 2015). Similarly, in tomato the cosmopolitan predatory mirid *Nesidiocoris tenuis* (Reuter) (Hemiptera: Miridae) enables effective control of *B. tabaci* and the tomato borer *Tuta absoluta* (Meyrick) (Lepidoptera: Gelechiidae) (Calvo et al., 2012; Urbaneja et al., 2012; Pérez-Hedo and Urbaneja, 2015, 2016; Pérez-Hedo et al., 2017), an important invasive tomato pest detected for the first time in Spain in 2007 (Desneux et al., 2010).

It is widely known that plants respond to herbivory through several signal transduction pathways that are mediated by phytohormones. The accumulation in the plant of the main phytohormones related to plant defenses, the jasmonic acid (JA), the salicylic acid (SA), the abscisic acid (ABA), and the ethylene (ET), activates signaling cascades that regulate transcriptional responses. These defenses can cause the production of secondary metabolites and proteins that have toxic, repellent and/or anti-nutritive effects on herbivores (direct defenses) (Kant et al., 2015). Furthermore, when the production and release of plant volatiles (Herbivore Induced Plant Volatiles; HIPVs) are triggered they can modify the behavior of both phytophagous pests and their natural enemies (indirect defenses) (Paré and Tumlinson, 1999; Kessler and Baldwin, 2001; Dicke, 2009). Recently, some of these zoophytophagous predators have been found to activate the same defense mechanisms as those triggered by herbivorous arthropods (Halitschke et al., 2011; Pappas et al., 2015, 2016; Pérez-Hedo et al., 2015a,b; Bouagga et al., 2018a,b; Zhang et al., 2018).

The mirid *N. tenuis* is capable of inducing plant defenses in tomato due to its phytophagous behavior. In a previous study, we verified how the phytophagy of the predator *N. tenuis* activated the metabolic pathway of ABA and JA in tomato plants, which made them less attractive to the whitefly *B. tabaci* and more attractive to the whitefly parasitoid *Encarsia formosa* (Gahan) (Hymenoptera: Aphelinidae) (Pérez-Hedo et al., 2015b). In addition, we observed how the volatiles emitted by the *N. tenuis* punctured plants induced defenses in neighboring untouched plants by activating the JA pathway. This induction also resulted in the attraction of parasitoids by these intact plants that had not been exposed to *N. tenuis* (Pérez-Hedo et al., 2015b). Later, we were able to confirm that all stages of development of *N. tenuis* (from young nymphs to adults) are able to trigger these defensive responses (Naselli et al., 2016). However, we show not all zoophytophagous predators have the same ability to induce such responses in tomato plants. Tomato plants may have different degrees of attraction for pests and natural enemies depending on whether phytophagous behavior occurs, for example, by *N. tenuis*, *Macrolophus pygmaeus* (Rambur) or *Dicyphus maroccanus* Wagner (Hemiptera: Miridae) (Pérez-Hedo et al., 2015a). Thus, while plants punctured by *N. tenuis* are rejected by *B. tabaci* and *T. absoluta*, the phytophagy of *M. pygmaeus* and *D. maroccanus* has no effect on repellence in *B. tabaci* and in fact attracts *T. absoluta*. In contrast, the feeding activity of these three mirids results in the attraction of *E. formosa*. This fact could be elucidated by identifying the volatiles (HIPVs) involved in the defensive responses of tomato plants induced by *N. tenuis* and *M. pygmaeus*; in general, plants exposed to *N. tenuis* emitted more volatiles than plants exposed to *M. pygmaeus*, and the latter emitted more volatiles than intact plants. Furthermore, six green leaf volatiles (GLVs) together with the methyl salicylate were found to be repellent to *B. tabaci* and attractive to *E. formosa*, whereas no effect on *T. absoluta* was observed. Octyl acetate, which was only significantly present in plants exposed to *M. pygmaeus*, was significantly attractive for *T. absoluta*, repellent for *E. formosa* and indifferent to *B. tabaci* (Pérez-Hedo et al., 2018). Similarly, in sweet pepper the phytophagy of the anthocorid *O. laevigatus* and the mirids *N. tenuis* and *M. pygmaeus* also trigger defense responses in this crop (Bouagga et al., 2018a,b).

Pappas et al. (2015, 2016) demonstrated a reduction in oviposition and the subsequent performance of the two-spotted spider mite *Tetranychus urticae* Koch (Acari: Tetranychidae) by *M. pygmaeus*. These authors attributed the reduction in *T. urticae* performance to a consequence of direct defense induction mediated by *M. pygmaeus*. *M. pygmaeus*-punctured tomato plants were observed to increase locally and systematically the accumulation of transcripts and the activity of protease inhibitors that are known to be involved in plant responses, resulting in detrimental effects on the life history traits of *T. urticae*.

However, the effect of these *N. tenuis* mediated plant defenses on plant selection, development, and reproduction of *T. urticae* remains unknown. In this research, we evaluated the olfactory response of *T. urticae* females exposed to *N. tenuis*-punctured tomato plants, JA-deficient mutant tomato plants and intact tomato plants, for comparison, in a Y-tube olfactometer. Secondly, the oviposition of *T. urticae* was evaluated on *N. tenuis*-punctured tomato plants and on intact tomato plants. Thirdly, a greenhouse experiment was conducted to evaluate whether the defense induction mediated by *N. tenuis* had an effect on *T. urticae*. Finally, we used gene expression analysis to assess whether *N. tenuis* activated JA signaling pathways and increased accumulation of transcripts of two proteinase inhibitor II markers which are known to be involved in plant defense.

## Materials and Methods

### Plants and Insects

Tomato plants *Solanum lycopersicum* cv. Moneymaker, JA-deficient tomato mutants (def-1) and their respective near-isogenic wild type (cv. Castlemart) parental lines were used to determine the responses of *T. urticae* and *N. tenuis* to the distinct experimental treatments described below. Seeds were sown in soil. Two weeks after germination seedlings were individually transplanted into pots (8 cm × 8 cm × 8 cm). Plants were maintained undisturbed at 25 ± 2°C, with constant relative humidity of 65% ± 5% and a photoperiod of 14:10 h (light: dark). Pesticide-free tomato plants were used for the experiments at 4 weeks of age (approximately 20 cm high). *N. tenuis* was provided directly by Koppert Biological Systems, S.L. (Murcia, Spain) and *T. urticae* adults were obtained from a culture established at IVIA in 2011 originally collected from the region of La Plana (Castelló, Spain). Mites were maintained on tomato plants kept in a climatic chamber at 25 ± 2°C, and 65% ± 5% RH and 14:10 h (light: dark).

*Nesidiocoris tenuis*-punctured plants were obtained by exposing tomato plants to 20 fourth instar nymphs for 24 h in a 30 cm × 30 cm × 30 cm plastic cage (BugDorm-1 insect tents; MegaView Science Co., Ltd., Taichung, Taiwan). Naselli et al. (2016) demonstrated that *N. tenuis* nymphs had the same potential to induce plant defenses in tomatoes as adults. Therefore, to avoid, on one hand, induction of defenses by adult oviposition and on the other hand accumulation and hatching of eggs along with interference in performance experiments, nymphs were used to induce defenses instead of adults. All motile individuals were removed from plants before the beginning of each trial.

### Y-Tube Bioassays

A Y-tube olfactometer experiment was conducted to test the olfactory responses of *T. urticae* and *N. tenuis* females to tomato plants that were previously punctured by *N. tenuis* relative to intact plants; to JA-deficient tomato mutant *def-1* and its near-isogenic wild type (*wt*) parental line. The Y-tube olfactometer (Analytical Research Systems, Gainesville, FL, United States) consisted of a 2.4-cm-diameter Y-shaped glass tube with a 13.5-cm long base and two arms each 5.75 cm long (Pérez-Hedo and Urbaneja, 2015). Both side arms were connected via high-density polyethylene (HDPE) tubes to two identical glass jars (5 L volume) each of which were connected to an air pump that produced a unidirectional humidified airflow at 150 ml/min (Pérez-Hedo and Urbaneja, 2015).

A single individual female was introduced into the tube (entry array) and observed until she had walked at least 3 cm up one of the arms or until 15 min had elapsed. A total of 30–40 valid replicates for each species were recorded for each pair of odor sources. Each individual was tested only once. Females that did not choose a side arm within 15 min were recorded as “no-choice” and were excluded from data analysis. After recording five responses, the Y-tube was rinsed with soapy water then acetone and left to dry for 5 min. The odor sources were subsequently switched between the left and right side arms to minimize any spatial effect on choice. Three types of plants (intact, mutant, and punctured) were used only once to test the response of 10 females and then were replaced with new plants. The Y-tube experiment was conducted under the following environmental conditions: 23 ± 2°C and 60 ± 10% RH.

### *Tetranychus urticae* Oviposition Mediated by the Exposure of the Plants to *N. tenuis*

The oviposition of *T. urticae* was evaluated on 10 *N. tenuis*-punctured tomato plants and on 10 intact tomato plants (cv. Moneymaker). Each of the plants were isolated inside a plastic cage (60 cm × 60 cm × 60 cm) (BugDorm-2 insect tents) maintained in a climate chamber at 25 ± 2°C and 60–80% RH with a 14:10 h (light: dark) photoperiod. For each of the plant types, two fully expanded leaflets were selected on which approximately one clip-cage was gently placed (3 cm on diameter). Inside each clip-cage, 10 presumably mated females of *T. urticae* were released and left undisturbed for 48 h. After this time, the clip-cages were removed and the number of *T. urticae* eggs was counted.

### *Tetranychus urticae* Survival and Reproductive Performance Mediated by the Exposure of the Plants to *N. tenuis*

The experiment was conducted in a 40 m × 10 m greenhouse equipped with drip irrigation system located at IVIA in Moncada (Valencia, Spain). The greenhouse was accessed through a double door and was divided into 12 experimental cages, six for *N. tenuis*-punctured plants and six for intact plants. Each cage represented one replicate. Cages were screened with “anti-thrips” polyethylene mesh with 220 μm × 331 μm interstices and the floor was covered with a 2 mm thick woven white polyethylene ground cloth. Each experimental cage was 2.5 m × 2 m × 2.5 m (L × W × H) and was accessed by a separate door secured with a zipper. One Datalogger (model TESTO 175-H2, Amidata S.A., Madrid, Spain) was placed in a central cage to record temperature and relative humidity. The average temperature during the experiment ranged between 23.5°C on the 31st of May, 2017 and 25.7°C on the 14th of June, 2017 with a minimum and maximum temperature of 20.4 and 34.8°C, respectively.

Eight tomato plants (cv. Moneymaker) were introduced into each cage. To avoid spider mite movement from plant to plant, plants were individually isolated, without touching either each other or the cage walls. Additionally, plants were placed on top of a brick inside a plastic tray full of water, and all pots and drip lines were painted with a band of glue. Plants were artificially infested with *T. urticae* from the previously mentioned laboratory population. Twenty *T. urticae* females were released per plant, distributed equally throughout the leaves with the aid of a fine brush (24th May, 2017). Seven and 14 days after *T. urticae* release samplings were conducted. Samplings involved counting the total number of *T. urticae* females on each plant. This was done with the naked eye, *in situ*, without removing leaves from the plant.

### Plant Gene Expression

The transcriptional response of the PIN2 wound-induced proteinase inhibitor II precursor (PIN2), a marker gene for JA, and two plant Proteinase Inhibitor II (PI-II1 and PI-II2) markers were studied on six *N. tenuis*-punctured tomato plants and on six intact tomato plants (Lopez-Raez et al., 2010; Pappas et al., 2015). The apical part of the tomato plant samples were immediately ground in liquid nitrogen. Portions of the ground samples were used for RNA extraction. Total RNA (1.5 μg) was extracted using a Plant RNA Kit (Omega BioTek Inc., Doraville, GA, United States) and was treated with RNase-free DNase (Promega Corporation, Madison, WI, United States) to eliminate genomic DNA contamination. The RT reaction and the PCR SYBR reaction were performed as described by Pérez-Hedo et al. (2015b). Quantitative PCR was carried out using the LightCycler^®^ 480 System (Roche Molecular Systems, Inc., Basel, Switzerland) sequence detector with standard PCR conditions. Expression of EF1 was used for normalization as a standard control gene. The nucleotide sequences of the gene specific primers are described in **Table 1**.

**Table 1:** Forward and reverse sequences of *PIN2* (Wound-induced proteinase inhibitor II precursor) marker gene for JA, PI-II1, and PI-II2 markers of plant proteinase inhibitor II, and the constitutive gene EF1.

Primers	Forward	Reverse
*PIN2*	5′-GAAAATCGTTAATTTAT CCCAC-3′	5′ -ACATACAAACTTTCCAT CTTTA-3′
PI-II1	5′-CATCATGGCTGTTCA CAAGG-3′	5′ -ATCCCGAACCCAAGAT TACC-3′
PI-II2	5′-GGCCAAATGCTTGCAC CTTT-3′	5′ -CAACACGTGGTACATC CGGT-3′
*EF1*	5′-GATTGGTGGTATTGG AACTGTC-3′	5′ -AGCTTCGTGGTGC ATCTC-3′

### Data Analysis

Chi-square (χ^2^) goodness of fit tests based on a null model were used to analyze data collected from the olfactory responses where the odor sources were selected with equal frequency. Individuals that did not make a choice were excluded from the statistical analysis. Two-tailed Student’s *t*-test (*P* < 0.05) was performed to compare oviposition between the two treatments and to compare the quantified expression of defense genes between intact plants and *N. tenuis*-punctured plants. Two measurements on two sample dates (7 and 14 days after *T. urticae* infestation) were analyzed using a generalized linear mixed model (GLMM) with repeated measures. Treatment was considered as a fixed factor and replicates nested within treatment was used as random factor to correct for pseudoreplication. The GLMM used a Poisson distribution with the logarithm as the link function. Results are expressed as the mean ± standard error.

## Results

### *N. tenuis*-Punctured Plants Do Not Alter *T. urticae* Plant Selection

**FIGURE 1:**
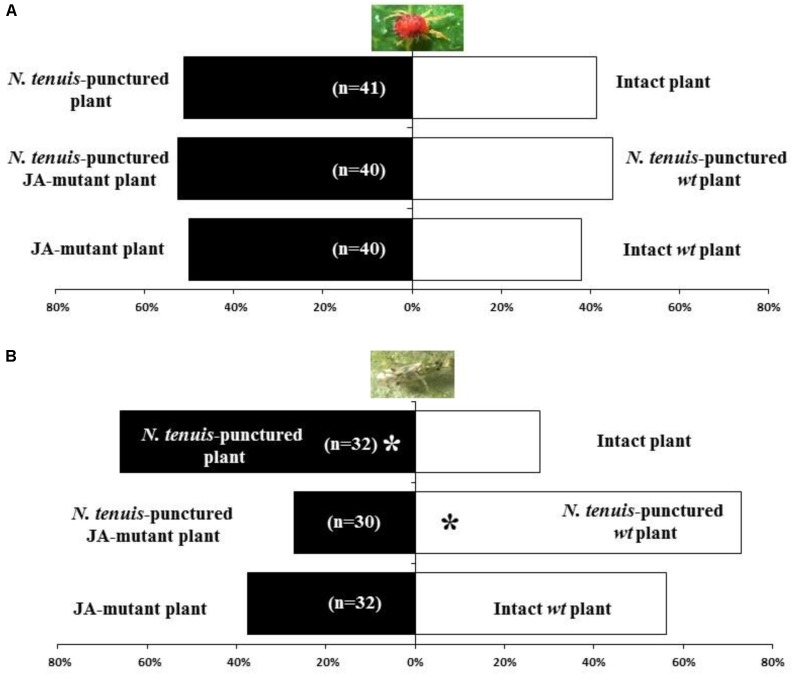
Response of the herbivore *T. urticae* females **(A)** and the natural enemy *Nesidicoris tenuis* **(B)** in a Y-tube olfactometer when exposed to JA-deficient mutant tomato plants or their near isogenic wild type (wt plant), which were with the zoophytophagous *Nesidiocoris tenuis* (*N. tenuis*-punctured plants) or without (intact plants) contact with *N. tenuis*. Significant differences using a χ^2^ test are marked with ^∗^*P* < 0.001.

The two-spotted spider mite, *T. urticae*, showed no preference for the odor source emitted by intact tomato plants when compared with *N. tenuis*-punctured plants. (χ^2^ = 0.842; *P* = 0.1794) (**Figure 1A**). Similarly, *T. urticae* had no preference when given a choice between the JA-mutant tomato plants and their near-isogenic *wt* that had and had not been exposed to mirids (χ^2^ = 0.461; *P* = 0.248; χ^2^ = 1.429; *P* = 0.116, respectively) (**Figure 1A**).

The mirid *N. tenuis* clearly chose *N. tenuis*-punctured plants when given a choice between intact plants and *N. tenuis*-punctured plants (χ^2^ = 9.600; *P* = 0.001; **Figure 1B**). The *N. tenuis*-punctured *wt* plants were also preferred to JA-mutant tomato plants previously punctured by *N. tenuis* (χ^2^ = 13.07; *P* = 0.0002). The mirid did not show a significant preference (χ^2^ = 2.400; *P* = 0.0607) when given a choice between JA-mutant plants or intact *wt* tomato plants (**Figure 1B**).

### *N. tenuis*-Punctured Plants Do Not Affect *T. urticae* Oviposition

**FIGURE 2:**
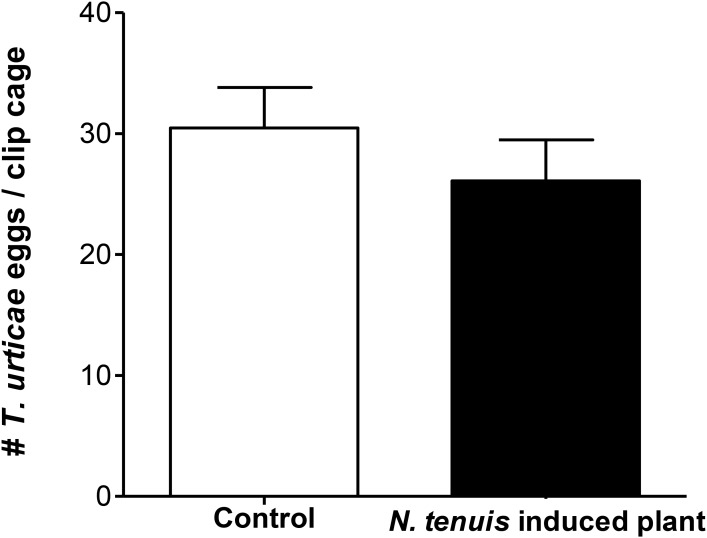
Number of eggs (mean ± SE) laid by 10 *T. urticae* females in a clip cage in 48 h on *N. tenuis*-punctured tomato plants and on intact tomato plants at 25 ± 2°C and 14:10 h L:D.

The number of eggs laid by *T. urticae* within each clip cage during 48 h was not significantly different when the females laid the eggs on intact tomato plants or on *N. tenuis*-punctured plants (*t* = 0.9165; df = 1, 36; *P* = 0.3655) (**Figure 2**).

### *N. tenuis*-Punctured Plants Reduce *T. urticae* Performance

**FIGURE 3:**
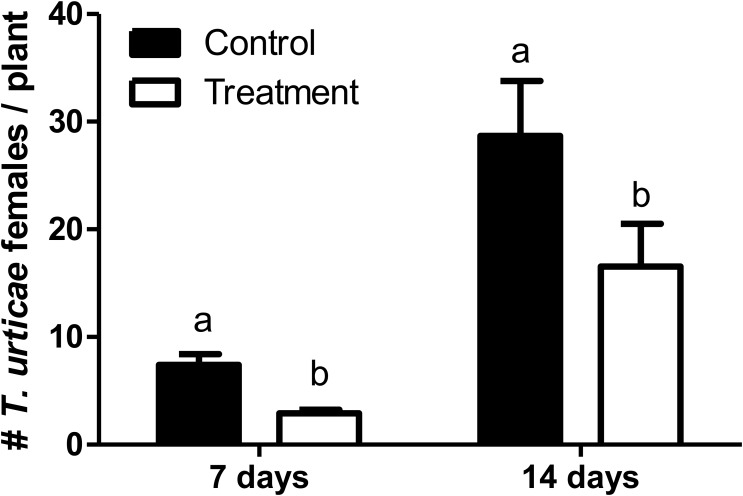
Number (mean ± SE) of *Tetranychus urticae* females per tomato plant in a glasshouse experiment comparing the mite development on *N. tenuis*-punctured tomato plants in comparison to intact tomato plants (Control). Bars with different letters are significantly different (GLMM, repeated measures; *P* < 0.05).

**FIGURE 4:**
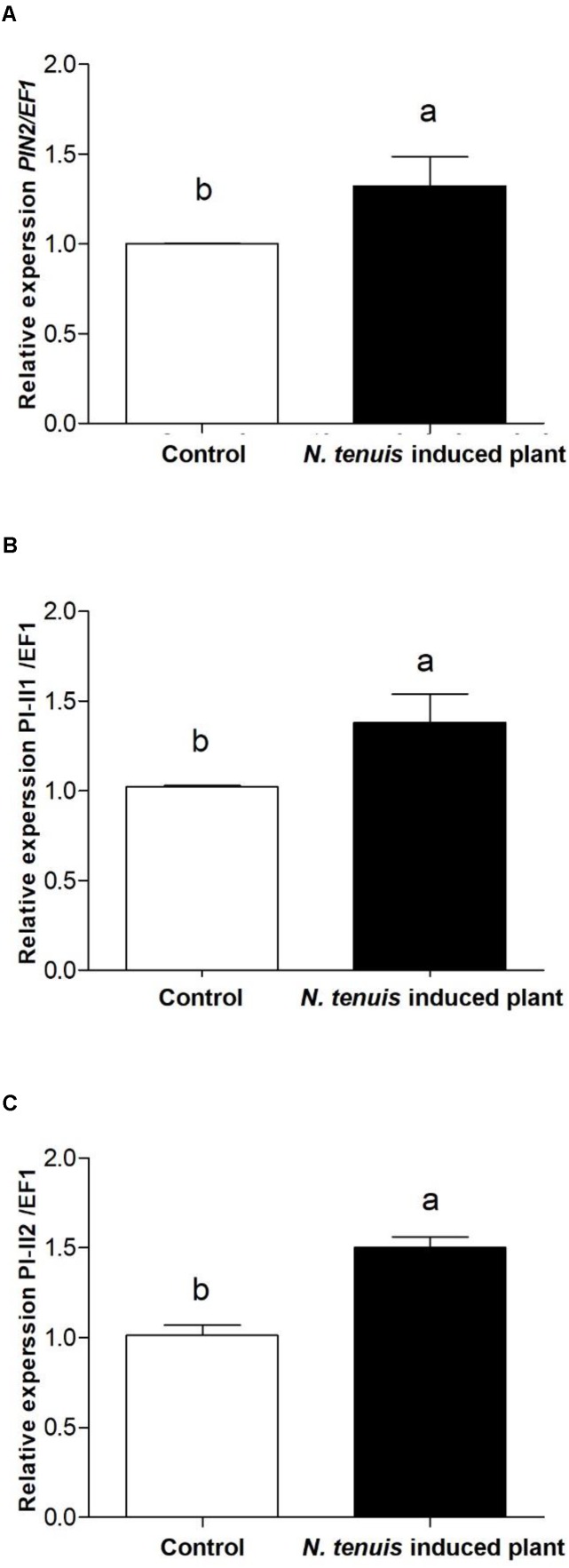
Transcriptional response of the defensive gene PIN2, a JA-responsive gene **(A)**, and two plant Proteinase Inhibitor (PI-II1 and PI-II2) markers **(B,C)**, in control tomato plants with comparison to plants pre-exposed to *N. tenuis* for 24 h. Data is presented as the mean of eight independent analyses of transcript expression relative to the constitutive EF1 gene ± SE (*n* = 8). Bars with different letters are significantly different (*t*-test; *P* < 0.05).

The number of *T. urticae* per plant was significantly lower in those tomato plants that were pre-exposed to *N. tenuis* (*F* = 16.612; df = 1, 166; *P* < 0.0001) (**Figure 3**). At day 14 the number of *T. urticae* per plant was significantly reduced by 35% on those plants previously activated by the feeding punctures of *N. tenuis* when compared to the control. The JA was significantly up-regulated and the concentration of protein inhibitors was higher on activated plants relative to the control (**Figure 4**). The analysis of the relative expression genes involved in indirect defense showed transcriptional differences between *N. tenuis*-punctured plants and intact tomato plants. The PIN2 gene (a marker for JA) was significantly up-regulated (*t* = 2.344; df = 10; *P* = 0.043) and the concentration of two plant protein inhibitors (PI-II1 and PI-II2) was higher on activated plants relative to the control (*t* = 2.260; df = 10; *P* = 0.047 and *t* = 5.924; df = 10; *P* < 0.0001, respectively) (**Figure 4**).

## Discussion

We have verified how *N. tenuis* is the activator of direct defense mechanisms responsible for reducing the performance of a key tomato pest such as the spider mite *T. urticae*. However, a clear effect on the *T. urticae* female choice mediated by HIPV’s by an *N. tenuis*-induced or by intact plant was not illustrated. It is known that the two-spotted spider mite uses odors to locate or avoid plants. Pallini et al. (1997) demonstrated that spider mites were weakly but significantly attracted to cucumber plants infested with conspecific herbivores, whereas strongly repelled by cucumber plants with heterospecific herbivores (i.e., the thrips *F. occidentalis*). Contrarily, Dicke (1986) observed that *T. urticae* dispersed when exposed to the odors of bean plants infested with spider mites. However, in our work *T. urticae* showed no repellence or attraction to the volatiles emitted by the defensive induction of *N. tenuis* which are mediated by the activation of the JA pathway. This conclusion was further confirmed with the use of JA-mutant plants, with and without previous punctures by *N. tenuis*, on which no response of *T. urticae* was obtained either. Therefore, in view of our results it seems that *T. urticae* does not respond to the volatiles induced by the phytophagy of *N. tenuis* through the JA pathway. These divergent results could be explained by the different composition of the volatile blends of each particular experimental situation aforementioned. Bouagga et al. (2018a) and Pérez-Hedo et al. (2018) showed that both of the mirid predators, *M. pygmaeus* and *N. tenuis* activated the JA pathway due to their phytophagous behavior in both of the crops, tomato, and sweet pepper. However, the composition of the volatile blend was specific at the species and plant level. In this work, through the use of JA-mutant plants, we demonstrate that the signaling pathway of JA is responsible for the attraction of the predator *N. tenuis*. Lins et al. (2014) previously illustrated that plants previously exposed to *N. tenuis* resulted attractive to *N. tenuis*. Pappas et al. (2015) observed that *T. urticae* deposited a lower number of eggs on plants previously exposed to the zoophytophagous predator, *M. pygmaeus*. Indeed, these authors found that this fecundity reduction was dependent on the predator density. However, in our study the oviposition of *T. urticae* was not affected when the mite was left undisturbed to lay eggs on either of the plants activated by *N. tenuis* or on intact plants. The methodology employed in our study and the one by Pappas et al. (2015) was quite different such as different mirid species, different time of mirid exposition and different number of mirids used to induce plants so the differences obtained between the studies could be due to the distinct way the mirid species, *N. tenuis* and *M. pygmaeus* activated the plants. Similar to our results, Ament et al. (2004) found that *T. urticae* laid as many eggs on JA-mutant plants as on wild-type plants.

Several previous studies demonstrated and explained the relationship between the activation of the JA pathway and the reduction in *T. urticae* performance (Arimura et al., 2000; Gols et al., 2003; Kant et al., 2004; Pappas et al., 2015) and even come to show that *T. urticae* infests and reproduces much better in JA-mutant plants than in wild plants (Li et al., 2002; Ament et al., 2004). Ament et al. (2004) suggested that JA-dependent direct defenses enhanced egg mortality or increase the time needed for embryonic development. In our research, *T. urticae* infestation was significantly lower in those plants that had been previously activated by *N. tenuis*. The activation of *N. tenuis* resulted in an up-regulation of the defensive gene PIN2, a JA-responsive gene, and two plant Proteinase Inhibitor (PI-II1 and PI-II2) markers. Pappas et al. (2015) already suggested that the decreased performance of *T. urticae* could be attributed to the higher concentration of PI in the induced plants by *M. pygmaeus* as occurred in our case with *N. tenuis*. Despite this and the effect of these PIs on other agricultural pests such as *Liriomyza trifolii* (Burgess) (Diptera: Agromyzidae) (Abdeen et al., 2005) or *Heliothis obsoleta* (Fabricius) (Lepidoptera: Noctuidae) (Abdeen et al., 2005) and bacterial diseases such as *Pseudomonas syringae* pv Tomato (Zhang et al., 2012), the exact role they play in the digestive physiology of phytophagous mites has yet to be clarified (Rehman et al., 2017).

The widespread use of *N. tenuis* in tomato greenhouses in southeastern Spain has ensured less pest pressure as well as fewer diseases in those crops where the mirid is well established (Calvo et al., 2012; Urbaneja et al., 2012; Pérez-Hedo and Urbaneja, 2016). The results of this study could partly explain how the incidence of *T. urticae* in crops where *N. tenuis* is being used is lower. Analogously, the direct induction triggered by the feeding punctures of *N. tenuis* could be affecting other key pests in this crop such as the whitefly *B. tabaci* and the lepidopteran *T. absoluta*. Preliminary results of our group suggest that plants activated by *N. tenuis* would also reduce the performance of both pests. Even more interesting would be to relate the activation of defenses and specifically the activation of the jasmonic pathway with the lower incidence of viruses. Since the use of zoophytophagous predators in horticultural crops has been promoted, lower incidence of some phytopathogenic viruses has been observed (Téllez et al., 2017). Recently, Escobar-Bravo et al. (2016) have shown that tomato plants with high expression of methyl jasmonate are less infected with the tomato yellow leaf curl virus (TYLCV). This led us to hypothesize that the defenses induced by *N. tenuis* in tomato could be altering the acquisition and multiplication of phytopathogenic viruses. However, further research is required to confirm this novel hypothesis.

It has been more than two decades since the activation of the jasmonic route has been shown to reduce the incidence of agricultural pests. Field studies have shown the application of exogenous JA to plants leads to a reduction in herbivore abundance and performance (Thaler, 1999) and increases plant fitness (Baldwin, 1998). Gols et al. (2003) also obtained a repellent effect for *T. urticae* when treating Lima bean plants directly with JA. The application of exogenous JA to cotton plants reduced spider mite oviposition rates by more than 75% (Omer et al., 2001). However, as far as our knowledge is concerned, this defense activation has not been put into practice nor adopted by growers for the improvement of pest management, except for the activation by zoophytophagous predators. With the widespread use of omic techniques and the increasingly vertiginous breakdown of the gene editing technique (CRISPR-Cas9), we think that the activation of defenses in plants will become a key tool for sustainable control of agricultural pests and diseases. To this end, our results can serve as a basis for the new management development of strategies for *T. urticae*, based on resistance mechanisms induced from the phytophagous behavior of *N. tenuis*.

## Author Contributions

MP-H and AU conceived and designed the research. All authors participated in data collection and analyses, wrote the manuscript, and read and approved the manuscript.

## Conflict of Interest Statement

The authors declare that the research was conducted in the absence of any commercial or financial relationships that could be construed as a potential conflict of interest.
